# Phylogenetic Analysis and In Vitro Antimicrobial Potential of Some Herbal Extracts Against Multidrug‐Resistant *Pseudomonas aeruginosa* Isolate Recovered From Hospital Wastes

**DOI:** 10.1155/jotm/4668333

**Published:** 2026-06-30

**Authors:** Muhammad Nazir Uddin, Taha Fatima, Mian Syed Ali Shah, Ishaq Khan, Wajid Khan, Saeedah Musaed Almutairi, Mohamed Soliman Elshikh, Farman Ullah, Iftikhar Ali, Adnan Shahzad, Taj-ud-Din

**Affiliations:** ^1^ Center for Biotechnology and Microbiology, University of Swat, Mingora, Pakistan, uswat.edu.pk; ^2^ Department of Botany and Microbiology, College of Science, King Saud University, P.O. 2455, Riyadh, 11451, Saudi Arabia, ksu.edu.sa; ^3^ Department of Life Sciences, Western Caspian University, Baku, Azerbaijan, wcu.edu.az; ^4^ College of Animal Science and Technology, Shandong Agricultural University, Taian, 271018, China, sdau.edu.cn; ^5^ Department of Genetics and Development, Columbia University Irving Medical Center, New York, 10032, New York, USA, columbia.edu; ^6^ Institute of Chemical Sciences, University of Swat, Mingora, Pakistan, uswat.edu.pk

**Keywords:** antimicrobial agents, extraction solvent system, *Myrtus communis*, *Pseudomonas aeruginosa*, *Punica granatum*

## Abstract

The emergence of multidrug‐resistant (MDR) *Pseudomonas aeruginosa* in healthcare settings poses a critical global health challenge, intensified by limited therapeutic options and the environmental spread of pathogens through hospital waste. This study addresses the urgent need for alternative strategies by investigating the phylogenetic profile and antimicrobial resistance of *P. aeruginosa* isolated from hospital dry waste in Saidu Sharif, Pakistan, while evaluating the antibacterial and antioxidant potential of nine ethnomedicinal herbs. The isolate, identified via 16S rRNA sequencing, exhibited 99% homology to the MDR strain *P. aeruginosa* 610D6 reported from China, underscoring potential transboundary resistance dissemination. Antibiotic susceptibility testing revealed resistance to 14 of 21 tested agents, with retained efficacy only for imipenem, cefoperazone/sulbactam (25‐mm inhibition zones), and fosfomycin (24 mm). Ethanol extracts of *Punica granatum*, *Myrtus communis*, and *Olea ferruginea* demonstrated remarkable antipseudomonad activity (26‐mm, 26‐mm, and 25‐mm zone of inhibitions, respectively) and potent antioxidant properties (97.57%, 95.55%, and 96.85% DPPH scavenging). Notably, solvent polarity significantly influenced extraction yields and bioactivity, with ethanol. This work highlights the therapeutic promise of plant‐derived compounds against MDR pathogens while encouraging for One Health approaches to mitigate antibiotic resistance.

## 1. Introduction

Hospital waste, especially biomedical waste, harbors infectious pathogens, which pose serious threats to the environment and immune‐compromised individuals [1]. Medical waste is generally inhabited by a variety of etiological agents, such as viruses, bacteria, fungi, and parasites [2]. The most important bacterial pathogens of hospital‐acquired infection are *Pseudomonas aeruginosa, Escherichia coli, Staphylococcus aureus, Klebsiella spp*., and *Clostridium difficile* [3]. Among these bacteria, *P. aeruginosa* is of great importance due to its ubiquitous nature and resistance mechanisms. *P. aeruginosa* is a gram‐negative, ubiquitous bacterium that can harbor in hospital waste [4]. The nosocomial infections caused by *P. aeruginosa* in high‐risk groups are life‐threatening [5]. About 10%–11% of all nosocomial infections are caused by *P. aeruginosa* [6]. It is known to be the cause of 13.2%–22.6% of all hospital‐acquired infections in intensive care units (ICUs) [7]. *P. aeruginosa* can cause pneumonia, urinary tract infections (UTIs), bloodstream infections, and surgical site and skin infections in the setting of burn injuries [7].

Proper diagnosis of infectious agents is essential for their effective treatment and control [8]. Molecular characterization and sequencing of bacterial isolates provide an accurate and authentic knowledge about the identification of bacterial species, their evolution, genetic diversity, and antibiotic resistance [9]. In the list of a large number of molecular methods, 16S rRNA sequencing is a gold standard used to identify the species level and conduct a phylogenetic analysis of bacterial species [10]. Moreover, the molecular sequencing is helpful in tracing the international distribution of bacterial strains and the appearance of new strains resistant to antibiotics [11].

Treatment of multidrug‐resistant (MDR) strains of *P. aeruginosa* is a real challenge [12]. Centers for Disease Control and Prevention (AR Threat Report) declares that MDR *P*. *aeruginosa* poses serious health threats [13]. The bacteria exhibit resistance to several antibiotics including aminoglycosides, quinolones, and *β*‐lactams [14]. This growing threat needs for alternative therapeutics, as WHO has stated that “no action today means, no cure tomorrow.” Medicinal plant extracts can be used as an alternative therapeutic option to treat bacterial infections [15].

The escalating threat of resistance in *P. aeruginosa* isolates, especially those that develop in the hospital environment, underscores an urgent need for alternative therapeutic strategies. The current study investigates the phylogenetic study and in vitro antimicrobial capacity of selected medicinal herbs against MDR *P. aeruginosa*, aiming to identify the potent medicinal herbs for future drug development.

## 2. Methodology

### 2.1. Sample Collection and Initial Processing

Samples were collected from hospital dry waste at Saidu Teaching Hospital, Swat, and brought to the Microbiology Lab, Center for Biotechnology and Microbiology, University of Swat, Pakistan. The samples were then processed for the isolation of pathogenic bacteria following standardized microbial culture techniques. The pure culture was identified as *P. aeruginosa* by a series of tests, i.e., gram staining, triple sugar ion test, indole test, coagulase test, citrate, catalase, oxidase, and urease [16].

### 2.2. Ethical Considerations

The proposed research study was approved by the Ethical Research Committee (ERC), University of Swat, Pakistan (No. UoS/ORIC/2023/35).

### 2.3. Molecular Identification and Characterization of *Pseudomonas* Isolate

The molecular identification and characterization of *P. aeruginosa* was performed through 16S rRNA sequencing. Extraction of genomic DNA and PCR assay were performed in Biotechnology Lab, Center for Biotechnology and Microbiology, University of Swat. The genomic DNA was obtained using the QIA amp DNA Mini Kit (Qiagen kit, Germany) following manufacturer’s protocol. The DNA pellet was purified via a DNA purification kit (Gene JET Genomic; Thermo Fisher Scientific‐K0721). The concentration and purity of DNA were checked by measuring the OD_260_/OD_230_ and OD_260_/OD_280_. For 16S rRNA sequencing, first, the 16S rRNA gene of the genome was amplified through universal bacterial primer (27f/1492r; 5′‐AGAGTTTGATCCTGGCTCAG‐3′/5′‐CTACGGCTACCTTGTTACGA‐3′). The PCR of 25 μL contained a DNA template (5 μL), each forward and reverse primer with the volume of 1 μL, 12.5 μL of master mix, and 5.5 μL PCR‐grade water. The thermocycler program was adjusted for 40 cycles, consisting of the following steps: DNA denaturation for 1 min at 94°C; primer annealing for 30 s at 60°C; elongation for 1 min at 72°C; and the final elongation step of 10 min at 72°C. The amplified product was run on 2% agarose gel in gel electrophoresis for 60 min at 110 V. The sample was then subjected to 16S rRNA sequencing. Before sequencing, a purification kit (GFXTM PCR DNA and Gel Band Purification) was used to purify PCR products, following the manufacturer’s protocol. Cycle Sequencing Kit (BigDye Terminator v3.1) was used to perform sequencing. The final sequencing reaction volume was 10 μL, containing 2 μL of sequencing buffer (5 ) and sequencing primers (forward and reverse primers of 16s rRNA gene). Sequencing was performed two times for each primer with the optimized sequencing condition (96°C for 1 min, followed by 25 cycles of denaturation at 96°C for 10 s, 50°C for 5 s, and 60°C for 4 min). Next, ethanol precipitation was used for cleaning the sequencing products. Following precipitation, the sequencing product‐containing tubes were centrifuged for 30 min at 13,000 rpm. After discarding the supernatant, the pellets were washed with ethanol (70%) and then centrifuged at 13,000 rpm for 30 min. The samples were then treated with HiDi formamide and loaded on a DNA analyzer (ABI 3730) [17].

### 2.4. Sequence Analysis

Finch‐Tv software Version 1.4.0 was used to analyze the reverse and forward sequences of the 16S rRNA gene of the bacterial isolate. Sequences with similar homology to consensus sequences were searched using BLAST of the NCBI site. Mega software was used to perform multiple sequence alignment. The Tamura‐Nei‐based neighbor‐joining method was used in phylogenetic analysis, and the tree was constructed by MEGA X (Version 10.1.8).

### 2.5. Antibiotic Susceptibility Test by Kirby‐Bauer’s Method

The antibiotic susceptibility test of the isolate was performed in the Microbiology Lab, Center for Biotechnology and Microbiology, University of Swat, Pakistan, through Kirby‐Bauer’s disk diffusion method. Commercially available 21 antibiotics (Thermo Scientific) of different generations were tested according to the guidelines outlined in the Clinical and Laboratory Standards Institute (CLSI) document M100 [18]. The tested antibiotics were tobramycin (10 μg), amikacin (30 μg), pipemidic acid (20 μg), linezolid (30 μg), lincomycin (15 μg), imipenem (10 μg), cefoperazone/sulbactam (75/30 μg), cefpodoxime (200 μg), cephalexin (30 μg), cefotaxime (30 μg), cefradine (30 μg), ceftriaxone (30 μg), ceftazidime (30 μg), piperacillin/tazobactam (100/10 μg), penicillin (10 μg), amoxicillin/clavulanic acid (10 μg), fosfomycin (200 μg), azithromycin (15 μg), clarithromycin (15 μg), teicoplanin (30 μg), and nitrofurantoin (300 μg). The results were determined by measuring zones of inhibition in millimeters (mm).

### 2.6. Crude Extract Preparation of Medicinal Herbs

Different parts of nine medicinal herbs (*Myrtus communis, Fumaria indica, Mirabilis jalapa, Mentha longifolia, Berberis lyceum, P. granatum, Withania somnifera, Olea ferruginea,* and *Glycyrrhiza glabra*) were used in this study (Table 1). The selection of these plants was based on the reported literature, local practitioners, and their use by local villagers. Mature plant materials were purchased from the local markets. Identification of these plants was done by Prof Dr. Dastager (Department of Botany, University of Peshawar). The plant parts were cleaned, dried, and powdered using home blenders. The crude extract was prepared using four solvents with different polarities: ethanol, N‐hexane, ethyl acetate, and aqueous solvents. Seventy grams (70 gm) of plant powder was steeped in 200 mL of each solvent. The flasks containing solutions were kept in the shaker at a speed of 160–180 rpm for a week. Afterward, the mixture was doubly filtered through Whatman filter paper 84 and dried under reduced pressure using a rotary evaporator. Extract yields were noted. Four different concentrations of crude plant extracts were prepared, i.e., 1000 μg/mL, 2000 μg/mL, 3000 μg/mL, and 4000 μg/mL in dimethyl sulfoxide (DMSO) [19]. The minimal inhibitory concentration (MIC) and minimal bactericidal concentration (MBC) of the plant extracts were determined by following the standardized methodology of Hemeg et al. [20] with some modifications. The plant extracts were serially diluted, and concentrations of 20 μg/mL to 5000 μg/mL were tested in the microdilution experiment.

**TABLE 1:** Medicinal herbs used against *Pseudomonas aeruginosa*.

S. no	Scientific name	Local name	Parts used
1	*Mentha longifolia*	Jangli Podina	Leaves
2	*Berberis lyceum*	Kawary	Roots
3	*Punica granatum*	Narsawe	Bark
4	*Withania somnifera*	Ashwagandha	Leaves
5	*Olea ferruginea*	Zaitun	Stem
6	*Glycyrrhiza glabra*	Mulathi	Fruit
7	*Myrtus communis*	Manro	Leaves
8	*Fumaria indica*	Papra	Leaves
9	*Mirabilis jalapa*	Gule Badian	Fruit

### 2.7. Antibacterial Susceptibility Test of Medicinal Herbs

The well diffusion method was used to test the efficacy of medicinal herbs against the isolates of *P. aeruginosa* by following the methodology of Manandhar et al. [21] with some modifications. In detail, the medium plates were cultured with the standardized culture of *P. aeruginosa* isolated from hospital dry waste. The plant extracts (1000 μg/mL, 2000 μg/mL, 3000 μg/mL, and 4000 μg/mL) in DMSO (5% v/v concentration) were poured into 8‐mm wells of inoculated plates. A volume of 100 μL per well was added to each well. The zones of inhibition were measured in millimeters (mm). DMSO was used as a negative control, while imipenem was used as a positive control.

### 2.8. Fourier Transformed Infrared Spectroscopy

The active functional groups of the most effective medicinal plant extracts (*P. granatum, M. communis,* and *O. ferruginea*) were identified using Fourier transform infrared spectroscopy (FTIR) according to the standardized method of Chizoruo et al. [22]. The FTIR spectra of the extracts were obtained using an FTIR spectrophotometer in the range of 4000–400 cm^−1^. The spectra (Figure 1a, b, c and d) were analyzed for the presence of specific groups and their peak values (Table 2).

FIGURE 1(a) FTIR spectra of the *Olea ferruginea* (4 mg) ethanol extract. (b) FTIR spectra of the *Punica granatum* (3 mg) ethanol extract. (c) FTIR spectra of the *Myrtus communis* (3 mg) ethanol extract. (d) FTIR spectra of the *Olea ferruginea* (3 mg) ethanol extract.

**Figure:**
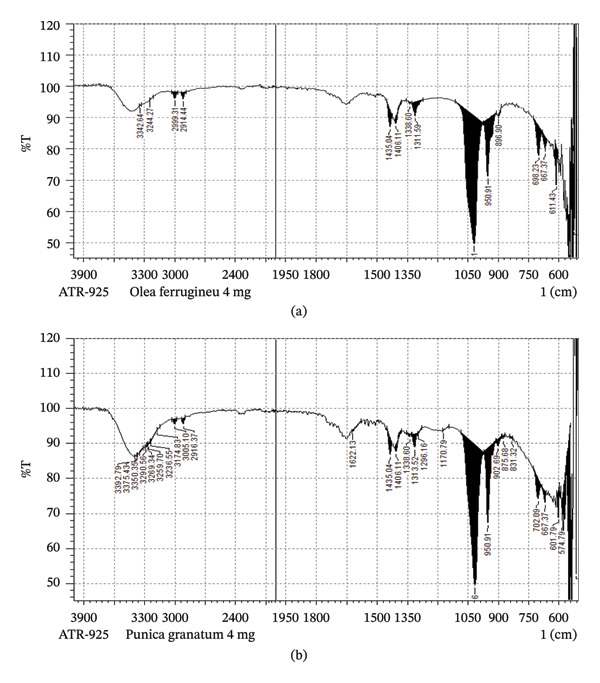

**Figure:**
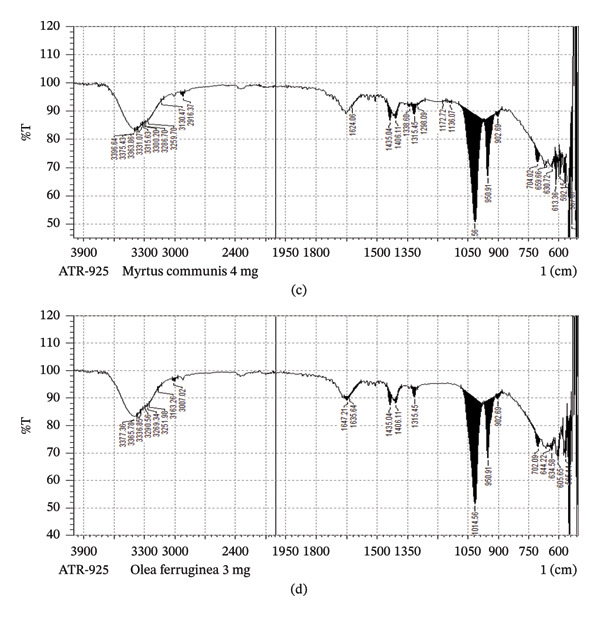

**TABLE 2:** FTIR analysis of effective medicinal plants.

Ethanol extract	Peak values ʋ (cm^−1^)	Functional groups
*Olea ferruginea*	3400–3244 cm^−1^, 2999 cm^−1^	ʋ(O‐H) stretching, ʋ(Ar‐H) sharp stretching,
	2914 cm^1^, 1650 cm^−1^	ʋ(R‐H) sharp stretching, ʋ(C=C) medium
	1435 cm^−1^, 1436 cm^−1^	Stretching, ʋ(C‐H) sharp bending, ʋ (C‐H)
	1359 cm^1^, 1047 cm^−1^	Sharp bending, ʋ(C‐H) sharp bending,
		ʋ (C‐H) sharp bending

*Punica granatum*	3400–3147 cm^−1^, 3009 cm^−1^ 2916 cm^−1^	ʋ(O‐H) stretching, ʋ(Ar‐H) sharp stretching,
	1622 cm^−1^, 1435–1313 cm^−1^, 1036 cm^−1^	ʋ(R‐H) sharp stretching, ʋ(C=C) medium
		Stretching, ʋ (C‐H) sharp bending, ʋ(C‐H)
		Sharp bending

*Myrtus communis*	4000–3259 cm^−1^, 3130 cm^−1^, 2916 cm^−1^	ʋ(O‐H) stretching, ʋ(Ar‐H) sharp stretching,
	1624 cm^−1^, 1435 cm^−1^, 1406 cm^−1^	ʋ(C=C) medium stretching, ʋ(C‐H) sharp
	1338 cm^−1^, 1315 cm^−1^,1056 cm^−1^	Bending, ʋ(C‐H) sharp bending, ʋ(C‐H)
		Sharp bending, ʋ(C‐H) sharp bending,
		ʋ(C‐H) sharp bending, ʋ(C‐H) strong stretching

*Olea ferruginea*	3400–3251 cm^−1^, 3007 cm^−1^	ʋ(O‐H) stretching, ʋ(Ar‐H) sharp stretching,
	2920 cm^−1^, 16,447‐1635 cm^−1^	ʋ(R‐H) sharp stretching, ʋ(C=C) medium
	1435 cm^−1^, 1408 cm^−1^, 1314 cm^−1^	Stretching, ʋ(C‐H) sharp bending, ʋ(C‐H) sharp
	1014 cm^−1^	Bending, ʋ(C‐H) sharp bending, ʋ(C‐H) sharp bending

### 2.9. Antioxidant Assay

The antioxidant potential of the effective medicinal plant extracts was determined using the 2, 2‐diphenyl‐1‐picrylhydrazyl (DPPH) assay. The assay was performed according to the standard protocol revised by Lalhminghlui and Jagetia [23]. The plant extract concentration was 400 μg/mL. The absorbance was measured using a UV–visible spectrophotometer. Ascorbic acid was used as a positive control and standard for comparing the antioxidant potential of extracts.

### 2.10. Data Analysis

All the experiments were performed in triplicate. The values were taken as mean of the triplicate experiment with standard deviation.

## 3. Results

The isolate was identified as *P. aeruginosa* by a series of tests, i.e., gram staining, triple sugar ion test, indole test, coagulase test, citrate, catalase, oxidase, and urease.

### 3.1. Molecular Identification and Characterization of *P. aeruginosa*

**FIGURE 2:**
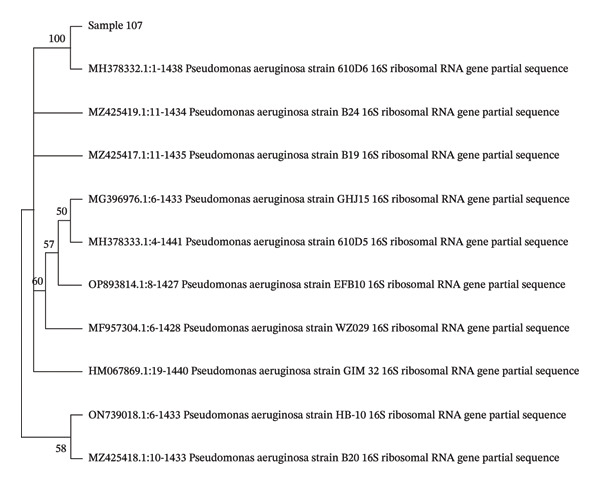
Phylogenetic presentation of *P. aeruginosa* isolated from hospital dry waste.

In the present study, an amplicon of ∼1450‐bp fragments was formed by the primer set of 16S rRNA when compared to the DNA marker. The amplicon of the isolate was subjected to direct sequencing using the same amplifying primers. The readable sequences were then blasted for the molecular identification and characterization of the isolate. The homology of the sequence with known sequences of gene bank was analyzed by different parameters (E value and percent identity). The phylogenetic tree was constructed on the bases of homology (Figure 2). The isolate showed 99% homology to *P aeruginosa*. The phylogenetic tree characterized the isolates into three clusters. Cluster 1 contains HB10 and B20 strains of *P. aeruginosa.* Cluster 2 contains five strains of *P. aeruginosa (*610D5, GHJ15, EFB10, WZ029, and GIM32*).* The isolate of *P. aeruginosa* recovered from medical waste was grouped with three strains of *P. aeruginosa* (610D6, B24, and B19) in Cluster 3. The isolate of *P. aeruginosa* from medical waste showed closer relation with *P. aeruginosa* 610D6.

### 3.2. Antibiotic Susceptibility of *P. aeruginosa*

**FIGURE 3:**
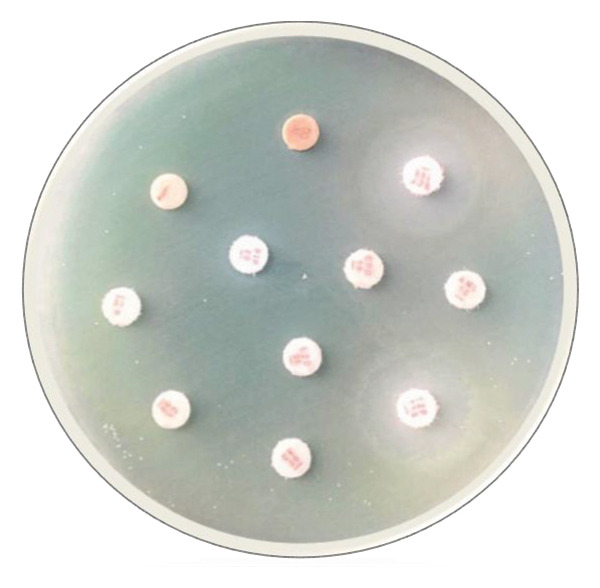
Antibiotic susceptibility of *Pseudomonas* *aeruginosa*.

In this study, the bacterium *P*. *aeruginosa* was noted as MDR and showed resistance to multiple commercially available antibiotics (Figure 3). The results of the antibiotic susceptibility showed that *P. aeruginosa* was susceptible to imipenem (25 mm), cefoperazone/sulbactam (25 mm), fosfomycin (24 mm), piperacillin/tazobactam (20 mm) and tobramycin (19 mm), amikacin (16 mm) and azithromycin (15 mm) exhibited intermediate activity according to CLSI (2025) breakpoints against the *P. aeruginosa* [18]. The remaining antibiotics were found to be ineffective against the bacterium (Table 3).

**TABLE 3:** Antibiotic susceptibility of *Pseudomonas aeruginosa*.

Antibiotic groups	Antimicrobial agent generation		Disk potency (μg)	Zone of inhibition(mm)
Aminoglycosides	TOB	3^rd^	10	19
	AK	3^rd^	30	16

Pyridopyrimidine	PIP	1^st^	20	_ _
Oxazolidinones	LZD	1^st^	30	_ _
Lincosamide	L	1^st^	15	_ _
Carbapenem	IPM	3^rd^	10	25
Cephalosporin	SCF	3^rd^	75/30	25
	CPD	3^rd^	200	_ _
	CFX	1^st^	30	_ _
	CTX	1^st^	30	_ _
	CE	3^rd^	30	_ _
	CRO	3^rd^	30	_ _
	CAZ	3^rd^	30	_ _

Penicillin	TZP	3^rd^	100/10	20
	P	1^st^	10	_ _
	AMC	3^rd^	10	_ _

Phosphonic	FF	2^nd^	200	24
Macrolide	AZM	2^nd^	15	15
	CLR	2^nd^	15	_ _

Semisynthetic glycopeptide	TEC	2^nd^	30	_ _
Nitrofuran	NFN	4^th^	300	_ _

*Note:* TOB: tobramycin, AK: amikacin, PIP: pipemidic acid, LZD: linezolid, L: lincomycin, IPM: imipenem, SCF: cefoperazone/sublactum, CPD: cefpodoxime, CFX: cephalexin, CTX: cefotaxime, CE: cefradine, CR: ceftriaxone, CAZ: ceftazidime, TZP: piperacillin/tazobactam, P: penicillin, AMC: amoxicillin/clavulanic acid, FF: fosfomycin, AZM: azithromycin, CLR: clarithromycin, TEC: teicoplanin, NFN: nitrofurantoin.

### 3.3. Anti‐*Pseudomonas* potential of medicinal herb Extracts

The extract yield from different parts of the plants in different extraction solvents was different (Table 4). The findings of the study indicated that the ethanol solvent had maximum extraction yield for all medicinal herbs except for *M*. *longifolia* with high extraction yield in water. The data also showed that the extract yield in polar solvents was higher than in nonpolar solvents.

**TABLE 4:** Percent yield of extracts of medicinal herbs in different solvents.

Medicinal herbs	% Yield of extract			
	Ethanol	Water	Ethyl acetate	Hexane
*Mentha longifolia*	9.8	20	4.2	1.1
*Berberis lyceum*	16	4	6	0.9
*Punica granatum*	14	8	5	0.8
*Withania somnifera*	16	13	3.5	1
*Olea ferruginea*	11	9	6.5	0.15
*Glycyrrhiza glabra*	9.5	7	4	0.6
*Myrtus communis*	12	11	5	0.9
*Fumaria indica*	10	8	5	0.7
*Mirabilis jalapa*	19	16	5	4

**FIGURE 4:**
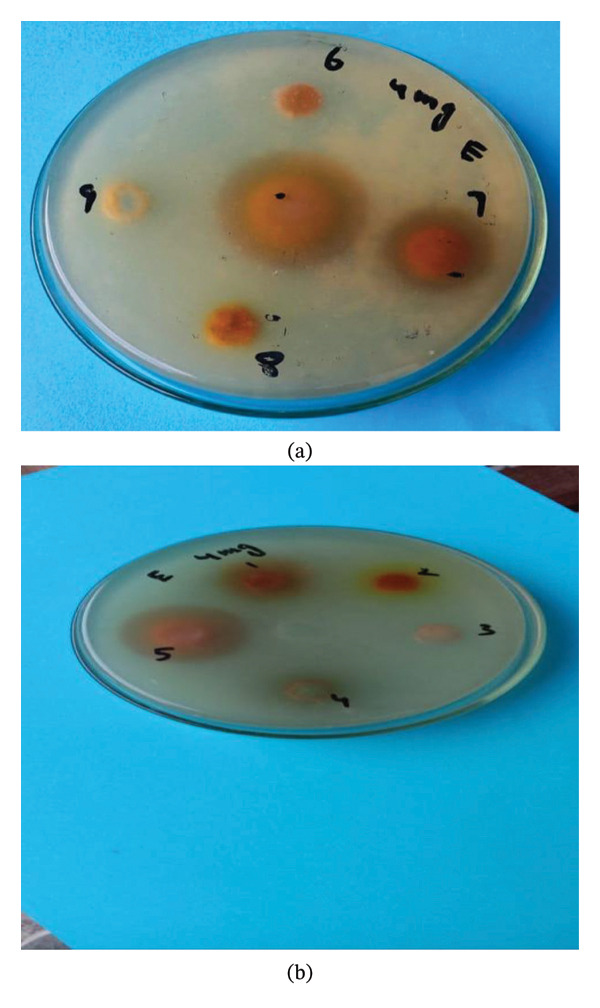
(a) and (b) Antibacterial potential of ethyl acetate extract of medicinal herbs against *P. aeruginosa*.

The extracts of medicinal herbs were tested against *P*. *aeruginosa* (Figure 4a and b). Strong antibacterial potential against the tested bacterium was found in the ethanol extract followed by water, n‐hexane, and ethyl acetate. The ethanol extracts of *P*. *granatum* (26‐mm ZI) and *M*. *communis* showed the highest antibacterial activity against *P. aeruginosa* and produced the same zone of inhibition (26 mm). *O. ferruginea* extracts exhibited significant results with a zone of inhibition of 25 mm. *W. somnifera* showed no antibacterial activity against the isolate (Table 5).

**TABLE 5:** Anti‐*Pseudomonas aeruginosa* activities of medicinal herbs.

Plants solvents	Zone of inhibition (mm) ± SD	1000 μg/mL	2000 μg/mL	3000 μg/mL	4000 μg/mL
*Mentha longifolia*	Ethanol	17 ± 0.10	8 ± 0.17	20 ± 0.15	20 ± 0.15
	Water	10 ± 0.20	10 ± 0.17	15 ±0.15	20 ± 0.15
	N‐hexane	_ _	12 ± 0.12	18 ± 0.12	20 ± 0.12
	Ethyl acetate	12 ±0.5	15 ± 0.5	15 ± 0.95	18 ± 0.5

*Berberis lyceum*	Ethanol	18 ± 0.19	17 ±0.12	17 ±0.12	18 ±0.10
	Water	15 ±0.10	15 ±0.10	18 ±0.10	20 ±0.5
	N‐hexane	10 ± 0.20	18 ±0.10	15 ±0.12	18 ±0.5
	Ethyl acetate	_ _	_ _	_ _	_ _

*Punica granatum*	Ethanol	20 ± 0.5	20 ±0.12	25 ±0.17	26 ±0.05
	Water	21 ±0.10	22 ±0.10	25 ± 0.9	25 ±0.5
	N‐hexane	20 ±0.12	20 ±0.13	25 ±0.17	25 ±0.17
	Ethyl acetate	20 ±0.05	20 ±0.12	25 ±0.12	25 ±0.15

*Withania somnifera*	Ethanol	_ _	_ _	_ _	_ _
	Water	_ _	_ _	_ _	_ _
	N‐hexane	_ _	_ _	_ _	_ _
	Ethyl acetate	_ _	_ _	_ _	_ _

*Olea ferruginea*	Ethanol	15 ± 0.12	25 ±0.15	22 ± 0.15	25 ±0.12
	Water	15 ±0.19	12 ±0.15	18 ±0.17	23 ±0.12
	18 ±0.12	22 ± 0.5	25 ±0.5	22 ±0.12	20 ±0.5
	N‐hexane	19 ±0.2	19 ±0.25	20 ±0.5	15 ±0.15

*Glycyrrhiza glabra*	Ethyl acetate	12 ±0.12	15 ±0.12	15 ±0.15	14 ±0.17
	Ethanol	_ _	12 ±0.5	12 ±0.5	_ _
	Water	_ _	_ _	_ _	_ _
	N‐hexaneEthyl Acetate	_ _	_ _	_ _	_ _

*Myrtus communis*	Ethanol	15 ±0.17	25 ±0.19	25 ±0.19	26 ±0.05
	Water	18 ± 0.15	20 ±0.10	20 ±0.13	23 ±0.3
	N‐hexane	16 ±0.13	16 ±0.12	20 ±0.13	22 ±0.13
	Ethyl acetate	_ _	_ _	_ _	_ _

*Fumaria indica*	Ethanol	15 ±0.5	20 ±0.10	22 ±0.12	22 ±0.13
	Water	18 ±0.17	20 ±0.17	20 ±0.19	23 ±0.5
	N‐hexane	16 ±0.5	16 ±0.10	20 ±0.10	22 ±0.12
	Ethyl acetate	_ _	_ _	_ _	_ _

*Mirabilis jalapa*	Ethanol	12 ±0.10	15 ±0.10	15 ±0.12	15 ±0.12
	Water	_ _	_ _	_ _	_ _
	N‐hexane	_ _	_ _	_ _	_ _
	Ethyl acetate	_ _	_ _	_ _	_ _

Abbreviation: SD = standard deviation.

Table 6 shows the values of the MIC and MBC of the nine medicinal plant extracts against *P. aeruginosa*. Great disparities were found in the plants and solvents tested. The ethanol extract of *M. communis* had the lowest MIC (50 μg/mL), and that of *P. granatum* and *O. ferruginea* possessed the next lowest MIC (100 μg/mL, ethanol and water extracts). *F. indica* also showed potent activity with MIC of 100 μg/mL in ethanol, water, and n‐hexane extracts. Conversely, *G. glabra* and *B. lyceum* exhibited higher MIC values (800–1000 μg/mL) particularly in nonpolar solvents.

**TABLE 6:** MIC and MBC of plant extracts against *Pseudomonas aeruginosa* isolates.

Plant	Solvent	MIC (μg/mL)	MBC (μg/mL)
*Mentha longifolia*	Ethanol	200 ±0.15	800 ±0.17
	Water	500 ±0.12	1000 ±0.10
	N‐hexane	–	–
	Ethyl acetate	800 ±0.5	2000 ±0.2

*Berberis lyceum*	Ethanol	1000 ±0.15	3000 ±0.17
	Water	1000 ±0.10	3000 ±0.3
	N‐hexane	2000 ±0.12	3000 ±0.17
	Ethyl acetate	–	–

*Punica granatum*	Ethanol	100 ±0.2	2000 ±0.10
	Water	100 ±0.3	2000 ±0.12
	N‐hexane	500 ±0.5	3000 ±0.2
	Ethyl acetate	1000 ±0.17	4000 ±0.3

*Withania somnifera*	Ethanol	–	–
	Water	–	–
	N‐hexane	–	–
	Ethyl acetate	–	–

*Olea ferruginea*	Ethanol	100 ±0.10	500 ±0.5
	Water	100 ±0.15	500 ±0.16
	N‐hexane	100 ±0.17	500 ±0.2
	Ethyl acetate	200 ±0.15	1000 ±0.2

*Glycyrrhiza glabra*	Ethanol	800 ±0.17	1000 ±0.5
	Water	–	–
	N‐hexane	–	–
	Ethyl acetate	–	–

*Myrtus communis*	Ethanol	50 ±0.3	100 ±0.2
	Water	100 ±0.2	200 ±0.15
	N‐hexane	100 ±0.10	200 ±0.17
	Ethyl acetate	–	–

*Fumaria indica*	Ethanol	100 ±0.4	800 ±0.15
	Water	100 ±0.16	800 ±0.12
	N‐hexane	100 ±0.15	800 ±0.12
	Ethyl acetate	–	–

*Mirabilis jalapa*	Ethanol	2000 ±0.10	4000 ±0.5
	Water	–	–
	N‐hexane	–	–
	Ethyl acetate	–	–

A similar trend was also seen with MBC results, with *M. communis* ethanol extract having the most significant bactericidal effect (100 μg/mL) followed by *O. ferruginea* having the uniform bactericidal effect at 500 μg/mL in ethanol, water, and n‐hexane. In contrast, *B. lyceum* required higher concentrates (3000 μg/mL) to kill the bacteria completely. It is interesting that MIC and MBC values could not be detected in *W*. *somnifera* extracts, and this fact indicates the lack of efficacy of these extracts against the analyzed *P. aeruginosa* strain.

### 3.4. Antioxidant Assay

The ethanol extracts, which showed strong antipseudomonads potential, were then subjected to an antioxidant assay. The results of the assay revealed that the ethanol extract of *P. granatum* has shown high radical‐scavenging activity (97.57%) followed by *O*. *ferruginea* (96.85%) and *M*. *communis* (95.55%) (Table 7).

**TABLE 7:** The antioxidant potential of ethanol extracts of medicinal herbs.

Extracts	Concentration (μg/mL)	% antioxidant activity
*Myrtus communis*	400 μg/mL	95.55
*Punica Granatum*	400 μg/mL	97.57
*Olea ferruginea*	400 μg/mL	96.85
Ascorbic acid	400 μg/mL	99.27

### 3.5. FTIR Assay

The FTIR spectra of ethanol extract of *M. communis, O. ferruginea*, *and P. granatum* exhibited a variety of structural features at different peeks. Extracts of these medicinal herbs revealed the presence of bioactive compounds through the FTIR spectra based on fingerprint traits and functional sets of peek positions. The predominant functional sets identified as C‐H (Aliphatic‐terpenes) at 2916 cm−1, C=O at 1730 cm−1 (Carbonyl‐Saponin), and at 1631 cm−1(resonating carbonyl of flavonoids), O‐H (polyphenols) at the 3284 cm−1, Peak at 1435 cm−1 showed presence of R‐O‐R (ether linkage). Cyclo‐hexyl bonding was detected in all extracts (Table 2 and Figure 1a, b, c).

## 4. Discussion

The current study supports the global spread of *P. aeruginosa* as a high‐risk pathogen [13], as the organism was found in biomedical waste and identified with the help of the biochemical test and the 16S rRNA gene, which has a better taxonomic accuracy and phylogenetic resolution [24]. Phylogenetic analysis revealed that there was a close association between the local isolate and previously reported strains in China, especially strain 610D6 (MH378332) of Nanjing, Jiangsu Province, and the least similarity was with strain B20 (MZ425418) of Jiangsu. It is important to note that strain 610D6 is reported to be antibiotic‐resistant [25], which creates the risk of common evolutionary changes.

The results of the antibiotic susceptibility test showed that cefoperazone/sulbactam and imipenem were the most effective agents, which are also consistent with previous studies [26, 27]. The activity of fosfomycin was also significant, which confirms the previous evidence of the correlation between the disk diffusion findings and the MIC values [28]. The intermediate resistance to tobramycin, amikacin, and azithromycin is in contrast with the studies that found the susceptibility to the agents [29, 30], which underscores the geographical and temporal variability of resistance patterns [31]. This variability is a sign of evolution and spread of resistance mechanisms based on environmental factors, hygiene, and prescriptions [32]. On the contrary, it was resistant to cephalosporins and penicillins, which was contrary to the previous clinical success of cephalosporins against *P. aeruginosa* in China [33]. The mutations of AmpC b‐lactamase are likely the cause of this resistance [34]. The isolate was also resistant to clarithromycin, teicoplanin, nitrofurantoin, pipemidic acid, linezolid, and lincomycin, which indicated a gradual change in the regional resistance profile. Antibiotic misuse and nonspecific prescription patterns may increase such trends [35]. This adaptability is further strengthened by established intrinsic and extrinsic mechanisms, such as biofilm formation and sustained cell development [35–37]. All these findings emphasize the necessity of constant monitoring and other treatment methods.

One of the evident tendencies in this work was the high reliance of the extraction yield and the antibacterial potency on the polarity of the solvent. Most medicinal herbs were always extracted most by ethanol except for *M. longifolia*, which was extracted best in water. Conversely, hexane produced the least amount of extracts, which is consistent with the available literature that polar solvents increase the extraction of bioactive compounds [38–44]. Ethanol extracts tended to yield the most potent antibacterial activity, especially *P. granatum*, *M. communis*, and *O. ferruginea*, than aqueous, n‐hexane, and ethyl acetate extracts. This trend indicates solvent‐dependent phenolics, tannins, and terpenoid extraction, which are commonly enriched in ethanol extracts and have antimicrobial activity [8, 45, 46]. The *P. granatum* ethanol extract had the best activity, which is supported by the earlier results of the high activity of pomegranate peel extracts [47]. The extracts of *O. ferruginea* were also effective with ethanol and n‐hexane extracts; these results are consistent with those of Hussain et al. [48]. *M. communis* was also highly inhibitory in ethanol, water, and hexane, which confirms the previous findings [49]. Other plants were more or less effective. *B. lyceum* aqueous extract demonstrated a 20‐mm zone, similar to the findings reported by Mughal et al. [50], and *F. indica* exhibited the greatest activity in water and the intermediate activity in ethanol and hexane, which is consistent with the previous data of the methanolic extract [51]. Conversely, *M. jalapa* was not very active in ethanol as it was reported before [52], which can be attributed to the differences in concentrations. *G. glabra* was intermediate in accordance with the result of Rafey et al. [53]. *W. somnifera* was not able to inhibit *P. aeruginosa* in the tested conditions. Altogether, the findings indicate a mechanistic relationship between the polarity of the solvent, the enrichment of phytochemicals, and the antibacterial activity. Ethanol is found to be the best solvent to extract bioactive antimicrobial metabolites.

An analysis of the most active ethanol extracts (*M. communis*, *O. ferruginea*, and *P. granatum*) by FTIR profiling identified functional groups of flavonoids, tannins, phenolics, carboxylic acids, and alkaloids. The functional groups of these bioactive compounds have already been reported in the previous literature of phytochemicals [54–56]. These functional signatures are especially associated with polyphenol‐linked and terpenoid‐linked functional signatures, which are strongly related to antimicrobial effects, including membrane disruption, enzyme inhibition, and metal chelation, as reported in previous studies [57–60]. The strong activity is probably due to the combined or synergistic effect of their actions [61, 62].

High radical‐scavenging capacities of *P. granatum*, *O. ferruginea*, and *M. communis* were also found in antioxidant assays, which are very close to those of ascorbic acid. The results suggest the presence of numerous phenolic and flavonoid compounds, which are molecules with dual antimicrobial and antioxidant effects [63]. Since the severity of infections is dependent on oxidative stress, antimicrobial and antioxidant extracts combined have beneficial and therapeutic effects [64]. Nevertheless, the mechanistic interaction between antioxidant capacity and anti‐*Pseudomonas* activity directly deserves further research.

MIC and MBC tests indicated that there was a significant difference between plant species and extraction solvents. *M. communis*, *P. granatum*, and *O. ferruginea* ethanol extracts exhibited the highest activity, and *M. communis* had especially low MIC/MBC values, which is an indication of effective bacteriostatic and bactericidal activity. These findings are consistent with the reports of the potent activity of pomegranate polyphenols [65], low MICs of ethanol peel extracts [66], and the strong activity of *O. ferruginea* [67]. On the other hand, *M. communis*, *G. glabra*, and *B. lyceum* have relatively higher potency requirements. The overall high quality of the ethanol extracts highlights the importance of polarity‐based extraction of phenolic and flavonoid compounds [40, 68]. The general tendency of increasing MBC compared to MIC is in line with the general plant‐derived antimicrobials in which the growth is usually inhibited at low concentrations, but total killing of bacteria is achieved at higher doses [69]. All these results highlight that ethanol extracts containing phytochemicals of *P. granatum*, *O. ferruginea*, and *M. communis* have a high potential to develop complementary or alternative therapeutic solutions against MDR *P. aeruginosa*.

## 5. Conclusion

The current study demonstrated that hospital waste of Saidu Sharif, Swat, is a source of MDR *P. aeruginosa*. The isolates were confirmed through biochemical tests and phylogenetic analysis of 16S rRNA gene sequencing. The antimicrobial assay of the isolates showed resistance to several commonly used antibiotics. These underscore the clinical challenges posed by MDR *P. aeruginosa* in local healthcare settings. The work further highlighted the potential of local medicinal herbs (*M. communis, P. granatum*, and *O. ferruginea*) as promising alternative therapeutic option to commonly used antibiotics. The major limitation of the research is the small sample size, which is limited in time, accessibility, and resources. Future studies must include broader sampling of different sites, isolate and characterize active constituents (i.e., punicalagins through HPLC), and test activity in biofilm models and in vivo systems. In addition, AmpC b‐lactamase mutations in environmental strains could be genomic screened to provide information about emerging resistance markers and inform regional antimicrobial stewardship initiatives.

## Author Contributions

Muhammad Nazir Uddin, Ishaq Khan, and Wajid Khan designed and supervised the study. Taha Fatima and Mian Syed Ali Shah performed the experiments. Saeedah Musaed Almutairi and Mohamed Soliman Elshikh performed the bioinformatics analysis and edited the manuscript. Farman Ullah, Taj‐ud‐Din, and Iftikhar Ali prepared the initial draft of the manuscript. Adnan Shahzad performed the analysis of FTIR data. Muhammad Nazir Uddin and Wajid Khan contributed to editing and revision of the manuscript.

## Funding

No funding was received for this manuscript.

## Conflicts of Interest

The authors declare no conflicts of interest.

## Data Availability

The data of the research study are available in the manuscript.
